# *TERT* C228T mutation in non‐malignant bladder urothelium is associated with intravesical recurrence for patients with non‐muscle invasive bladder cancer

**DOI:** 10.1002/1878-0261.12746

**Published:** 2020-06-27

**Authors:** Yujiro Hayashi, Kazutoshi Fujita, Satoshi Nojima, Eisuke Tomiyama, Makoto Matsushita, Yoko Koh, Kosuke Nakano, Cong Wang, Yu Ishizuya, Taigo Kato, Koji Hatano, Atsunari Kawashima, Takeshi Ujike, Motohide Uemura, Ryoichi Imamura, Eiichi Morii, Norio Nonomura

**Affiliations:** ^1^ Department of Urology Osaka University Graduate School of Medicine Suita Japan; ^2^ Department of Urology Kindai University Faculty of Medicine Osakasayama Japan; ^3^ Department of Pathology Osaka University Graduate School of Medicine Suita Japan; ^4^ Department of Urological Immuno‐oncology Osaka University Graduate School of Medicine Suita Japan

**Keywords:** bladder cancer, field change, prognosis, TERT promoter

## Abstract

Telomerase reverse transcriptase (*TERT*) promoter mutations are frequently found in tumors or urine from patients with urothelial carcinoma (UC). *TERT* promoter mutations are also detected in urine from patients with no evidence of cancer but are associated with subsequent UC development. Mutations in the *TERT* promoter are thought to be present in nonmalignant urothelium (NMU) during early stages of tumor formation prior to pathological change, but this has not been proven directly. In this proof‐of‐concept study, we investigated the clinical utility of *TERT* promoter mutation analysis in NMU of patients with non‐muscle‐invasive bladder cancer (NMIBC). This single‐institute study included 53 primary tumors and 428 systematic bladder biopsy specimens from 54 patients with NMIBC. All patients underwent systematic random biopsy and transurethral resection of the bladder tumor. Genomic DNA was analyzed for *TERT* C228T and C250T mutations using droplet digital PCR (ddPCR). The association between *TERT* promoter mutation of NMU and bladder recurrence was examined by the Kaplan–Meier method and Cox proportional hazards model. Of the 54 patients, 16 (29.6%) had a *TERT* C228T mutation and three (5.6%) had a *TERT* C250T mutation in NMU. Of 428 biopsy specimens, the *TERT* C228T mutation was detected in 9% (31/364) of normal urothelium, 27% (4/15) of urothelial dysplasia (UD), 50% (9/18) of UD suspicious for carcinoma *in situ* (CIS), and 58% (18/31) of CIS. During follow‐up (median: 3.7 years), 22 (40.7%) patients experienced bladder recurrence and five (9.3%) experienced disease progression. Cox proportional hazard analysis showed that *TERT* C228T mutation in NMU was significantly associated with bladder recurrence after adjustment for cofounding factors (*P* = 0.0128). Thus, *TERT* C228T mutation was detected in NMU, which was a reliable independent prognostic factor of bladder tumor recurrence.

## Graphical abstract

Telomerase reverse transcriptase (*TERT*) C228T mutation was detected in normal urothelium and urothelial dysplasia of patients with nonmuscle invasive bladder cancer (NMIBC) and may thus be a novel and useful prognostic factor for risk stratification of NMIBC. Analysis of systematic random biopsies for *TERT* promoter mutations in nonmalignant urothelium may guide selection of the optimal treatment strategy.

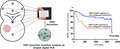

**Abbreviations**
BCG: Bacillus Calmette–Guérin
CI: confidence interval
CIS: carcinoma *in situ*
EORTC: European Organisation for Research and Treatment of Cancer
HR: hazard ratio
NMIBC: non‐muscle‐invasive bladder cancer
TERT: telomerase reverse transcriptase
TURBT: transurethral resection of bladder tumor
UBC: urothelial bladder cancer
UC: urothelial carcinoma

## 1 Introduction

Urothelial bladder cancer (UBC) is the most common malignancy of the urinary tract. Approximately 70% of patients with UBC have non‐muscle‐invasive bladder cancer (NMIBC) at initial diagnosis [1], which can be cured by a transurethral resection of bladder tumor (TURBT) and/or intravesical therapy. Although the overall survival of patients with NMIBC is generally good, 50–80% have intravesical recurrence after TURBT and 10–15% experience disease progression [2, 3]. For this reason, patients need to be monitored for recurrence at regular intervals for a couple of years using cystoscopy, urine cytology, and upper tract imaging. Currently, there are several risk stratification systems for predicting the probability of disease recurrence and progression, but these systems are not optimal for stratifying risk of disease recurrence or progression in patients with NMIBC [3, 4, 5, 6]. Therefore, novel risk stratification tools are needed in order to improve patient outcome.

Hotspot mutations of the telomerase reverse transcriptase (*TERT*) promoter are frequently identified in primary tumors from patients with various types of bladder cancer and tumor stages, including precancerous lesions [7, 8, 9, 10, 11, 12, 13]. Mutations in the upstream promoter of *TERT* predominantly affect two positions, g.1295228 C>T (C228T) and g.1295250 C>T (C250T) [14]. These mutations in the *TERT* promoter allele alter the binding site and subsequently result in increased *TERT* expression, which enables tumors to maintain their telomere length and avoid senescence [15]. Furthermore, *TERT* promoter mutations result in a gradual upregulation of telomerase, which contributes to tumorigenesis by promoting genomic instability [16]. The tumor mutation status of the *TERT* promoter, including *TERT* C228T and C250T, is associated with disease recurrence and progression of NMIBC [17, 18]. In contrast to other gene mutations frequently found in urothelial carcinoma (UC), such as tumor protein 53 (*TP53*) and fibroblast growth factor receptor 3 (*FGFR3*), *TERT* promoter mutations are persistently present in both primary and recurrent tumors of most patients with NMIBC [17].

We previously reported that *TERT* promoter mutations in urinary cell‐free DNA (cfDNA) and urinary cellular DNA could be detected prior to the tumors becoming macroscopically visible using a liquid biopsy technique [19, 20]. It is thought that important cancer‐initiating events such as driver gene mutations could occur in pathologically nonmalignant tissues as a consequence of ‘field change’ [21]. *TERT* promoter mutations were detected in precancerous lesions in patients with hepatocellular carcinoma or malignant melanoma [22, 23]. For these reasons, we hypothesize that cfDNA or urinary cellular DNA containing *TERT* promoter mutations might be released from precancerous cells with a nonmalignant appearance as a field change. In the current study, we examined the profile of *TERT* promoter mutations in nonmalignant urothelium (NMU) of systematic biopsy samples collected at the time of TURBT and analyzed the association of the mutations with intravesical recurrence of NMIBC.

## 2 Methods

### 2.1 Clinical samples

We randomly selected NMIBC patients who received TURBT and systematic bladder biopsy at Osaka University Hospital, Suita, Japan, from 2013 to 2018 and provided written informed consent. The study was approved by the Institutional Review Board of Osaka University (IRB #668‐3) and conformed with the Declaration of Helsinki. To exclude concurrent carcinoma *in situ* (CIS), systematic random biopsy was performed for all patients who received TURBT for the first time or who had positive urine cytology in our institution. We analyzed 428 systematic bladder biopsy specimens obtained from the trigone, posterior wall, bladder dome, anterior wall, right wall, left wall, bladder neck, or prostatic urethra (male only) and 53 primary tumors (one tumor was missing) from 54 patients with NMIBC.

### 2.2 Pathological diagnosis

Histological diagnoses were determined by at least two experienced pathologists. Biopsy specimens were diagnosed as normal urothelium (NU), urothelial dysplasia (UD), UD suspicious for CIS, or CIS. NMU was defined as either NU or UD as previously described [24]. Tumor stage and grade were determined according to the American Joint Committee on Cancer 8th Edition Cancer Staging Manual [25], and the tumors were graded according to the World Health Organization 2016 criteria [26].

### 2.3 DNA extraction

DNA extraction from the clinical specimens was performed using a GeneRead DNA FFPE Kit (Qiagen, Hilden, Germany) according to the manufacturer's protocol. All DNA extractions were performed using ten–fifteen 10‐µm sections from each biopsy sample and two or three 10‐µm sections from each tumor sample. DNA concentrations were measured using a Qubit dsDNA High Sensitivity Assay Kit (Thermo Fisher Scientific, Waltham, MA, USA).

### 2.4 Droplet digital PCR

For mutation detection, the QX100 ddPCR System (Bio‐Rad Laboratories, Hercules, CA, USA) along with primers and probes (FAM, mutant‐type, and HEX, wild‐type) and ddPCR Supermix for probes (No dUTP) from Bio‐Rad Laboratories were employed according to the manufacturer's protocol. Details on the primers and probes for *TERT* promoter mutants C228T and C250T used in the ddPCR analyses are shown in Table S1. Droplets were generated using a droplet generator (Bio‐Rad Laboratories). The PCR cycling parameters included 10 min at 95 °C, 50 cycles of 94 °C for 30 s and 55 °C for 1 min, and one cycle of 98 °C for 10 min followed by a 12 °C hold. Droplet fluorescence was assessed using a droplet reader. Analysis of the ddPCR data for allele calling and the calculation of absolute copy numbers were performed using quantasoft software, version 1.7.4 (Bio‐Rad Laboratories). The samples were considered true positive for targeted mutations when the following two criteria were met. First, they contained at least three droplets in the positive area of the FAM signal. Second, the mutant allele frequency (MAF) was > 5.0% to exclude false‐positive reactions induced by DNA degradation due to the fixation process. MAF was defined as the proportion of mutant‐type copies relative to the total number of copies including both mutant‐type and wild‐type alleles as determined by the ddPCR analyses.

### 2.5 Statistical analysis

Statistical analysis was performed using jmp pro 14.0.0 software (SAS Institute Inc., Cary, NC, USA). Cox regression analyses were performed to assess the relative contributions of various factors and *TERT* promoter mutations. Because of the limited sample size, we applied two multivariate models to investigate factors associated with bladder recurrence. Differences were considered statistically significant when the *P*‐value was < 0.05.

## 3 Results

### 3.1 *TERT* promoter mutations in nonmalignant urothelium

**Fig. 1:**
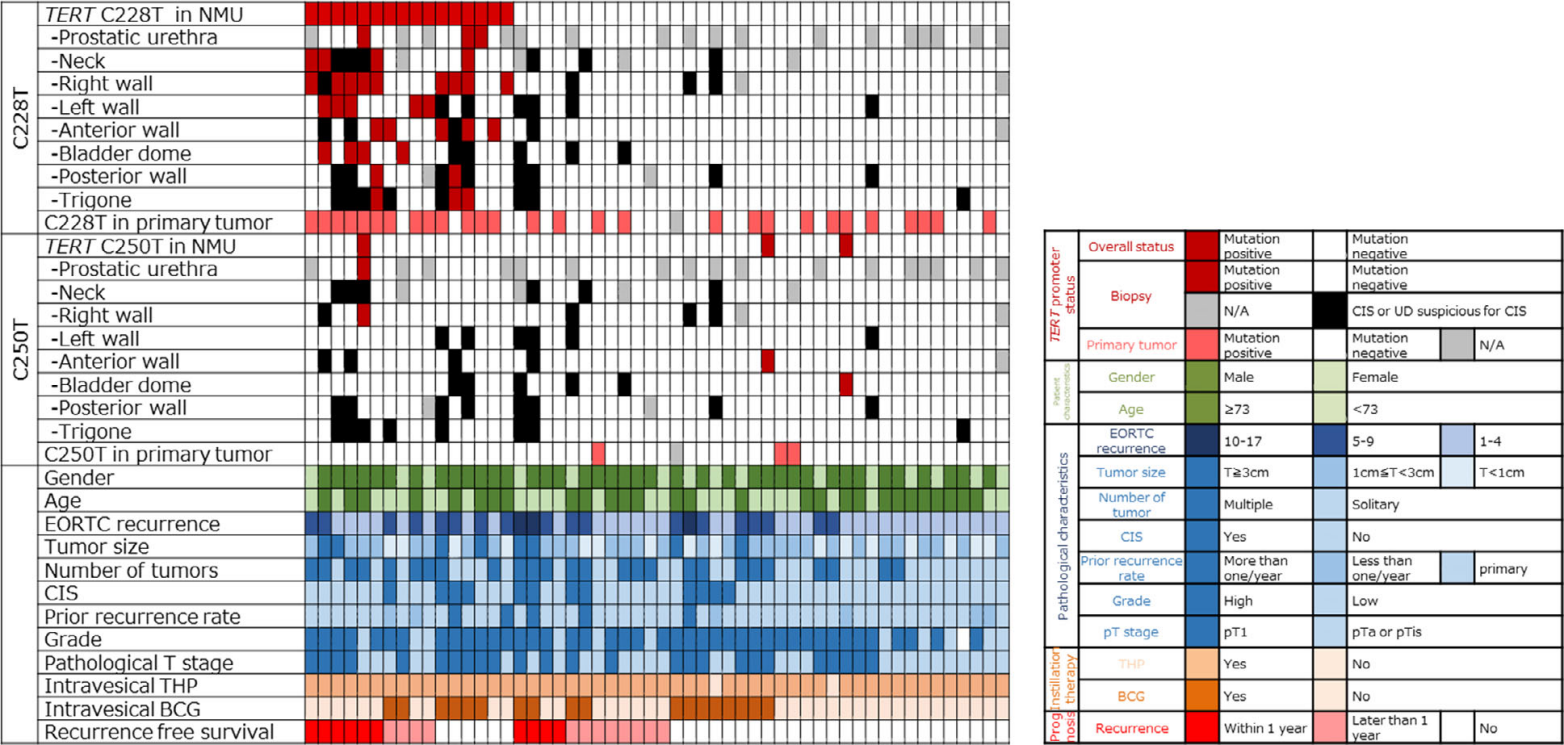
Association of clinical and pathological features and *TERT* promoter mutations. N/A, not applicable; THP, pirarubicin.

**Fig. 2:**
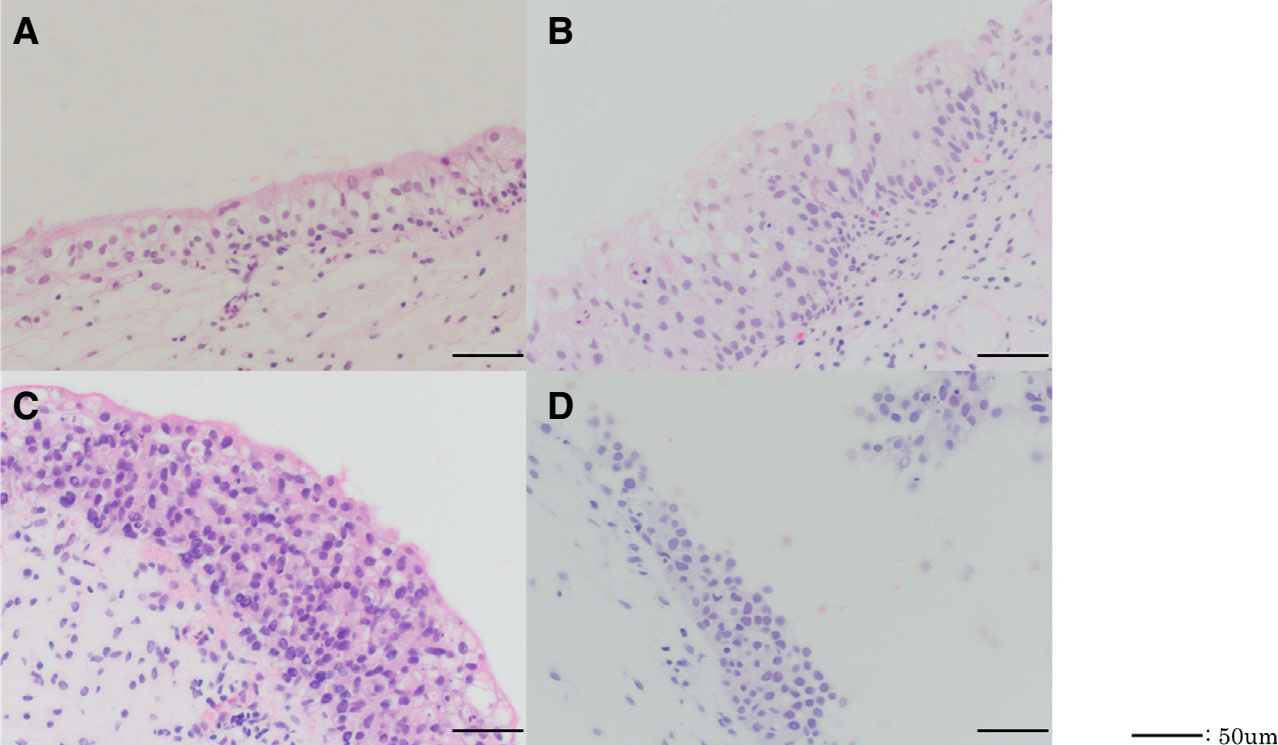
Representative hematoxylin and eosin staining of *TERT* C228T‐positive lesions. (A) NU. (B) Urothelial dysplasia. (C) Urothelial dysplasia suspicious for CIS. (D) CIS. Black bar indicates 50 μm.

Fifty‐four patients with NMIBC who underwent TURBT and simultaneous systematic random biopsy were included in the analysis. The median age of the patients was 73 years (range, 33–87). The association of clinicopathological characteristics and *TERT* promoter mutations is shown in Table 1 and Fig. 1. Among the 54 patients, 18 (33.3%) had *TERT* promoter mutations in NMU; 16 (29.6%) had a *TERT* C228T mutation; and three (5.6%) had a *TERT* C250T mutation. There was no difference in age between patients with and without *TERT* C228T mutation in NMU (*P* = 0.348). Among 28 patients with *TERT* C228T‐positive primary tumors, 13 (46.4%) were also *TERT* C228T‐positive in NMU. On the other hand, among 25 patients with *TERT* C228T‐negative primary tumors, three (12.0%) were *TERT* C228T‐positive in NMU. Representative microscopic images for each pathological diagnosis with the *TERT* C228T mutation are shown in Fig. 2. The frequency of *TERT* promoter mutations for each biopsy specimen and the primary tumors are shown in Table 2. Of 428 biopsy specimens, the *TERT* C228T mutation was detected in 9% (31/364) of NU, 27% (4/15) of UD, 50% (9/18) of UD suspicious for CIS, and 58% (18/31) of biopsy‐proven CIS.

**Table 1:** Characteristics of 54 patients with NMIBC.

	Overall (*n* = 54)	*TERT* C228T‐positive in NMU (*n* = 16)	*TERT* C228T‐negative in NMU (*n* = 38)
	No.	No. (%)	No. (%)
Gender			
Male	39	13 (33)	26 (67)
Female	15	3 (20)	12 (80)
Age, years			
Median (range)	73 (33–87)	72 (59–86)	73 (33–87)
Number of tumors			
Single	28	7 (25)	21 (75)
2–7	25	9 (36)	16 (64)
≥ 8	1	0 (0)	1 (100)
Tumor diameter			
< 3 cm	46	12 (26)	34 (74)
≥ 3 cm	8	4 (50)	4 (50)
Prior recurrence rate			
Primary	43	12 (28)	31 (72)
≤ 1 recurrence/year	6	2 (33)	4 (67)
> 1 recurrence/year	5	2 (40)	3 (60)
Pathological T stage			
pTa	26	6 (23)	20 (77)
pT1	25	10 (40)	15 (60)
Concurrent CIS	12	4 (33)	8 (67)
≥ pT2	0		
No malignancy	0		
Grade			
G1	0		
G2	27	8 (30)	19 (70)
G3	27	8 (30)	19 (70)

**Table 2:** Frequency of *TERT* promoter mutations in biopsy specimens and primary tumors.

	Biopsy specimens (*n* = 428)				Primary tumors (*n* = 53)	
	NU	UD	UD suspicious for CIS	CIS	pTa or pTis	pT1
*TERT* C228T mutation	9% (31/364)	27% (4/15)	50% (9/18)	58% (18/31)	52% (15/29)	54% (13/24)
*TERT* C250T mutation	1% (4/364)	0% (0/15)	0% (0/18)	0% (0/31)	10% (3/29)	0% (0/24)

### 3.2 Association of *TERT* promoter mutations with intravesical tumor recurrence

**Fig. 3:**
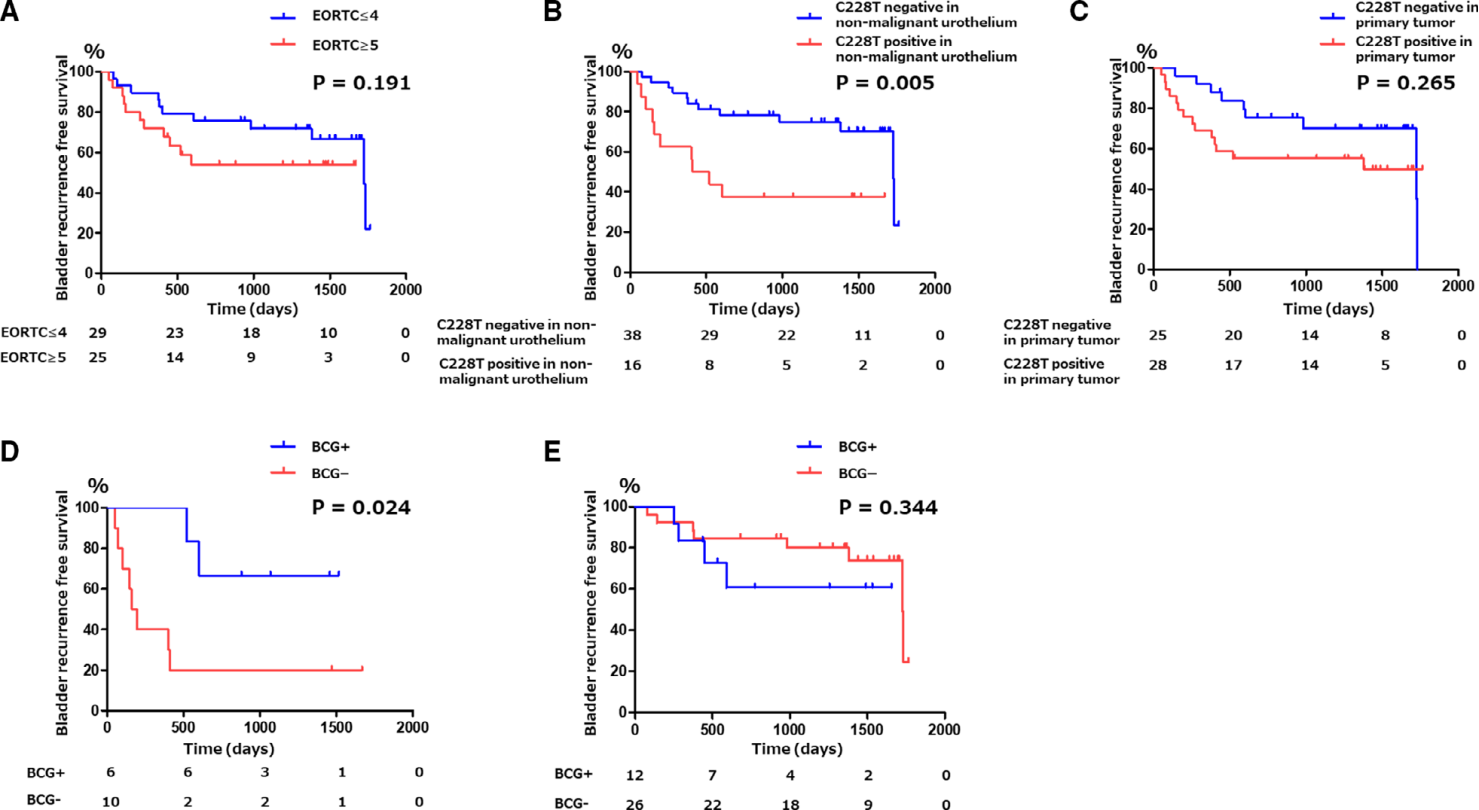
Kaplan–Meier curves for bladder recurrence‐free survival. (A) Stratified by EORTC recurrence score. (B) Stratified by *TERT* C228T mutation status in NMU. (C) Stratified by *TERT* C228T mutation status in primary tumors. (D) Stratified by BCG induction therapy for patients with *TERT* C228T mutation in NMU. (E) Stratified by BCG induction therapy for patients without *TERT* C228T mutation in nonmalignant urothelium.

The median follow‐up period was 3.7 years (range, 0.8–4.8). Based on the European Organisation for Research and Treatment of Cancer (EORTC) recurrence scoring system, 29 (53.7%) patients had a score of 1–4 (intermediate‐low risk), 20 (37.0%) had a score of 5–9 (intermediate‐high risk), and 5 (9.3%) had a score of 10 (high risk). Fifty‐three patients (98.1%) received immediate postoperative intravesical instillation therapy with pirarubicin and 18 (33.3%) received intravesical Bacillus Calmette–Guérin (BCG) induction therapy (Fig. 1). BCG maintenance therapy is not administered in our institution. BCG therapy was recommended for patients with CIS lesions or a pT1 tumor in our institution. During the follow‐up period, 22 (40.7%) patients experienced NMIBC recurrence and five (9.3%) experienced disease progression including muscle‐invasive disease or distant metastasis. There was no significant difference in the bladder recurrence rates among the groups according to the EORTC recurrence risk classification (Fig. 3A). Patients with the *TERT* C228T mutation in NMU had a significantly greater chance of bladder recurrence compared to that of patients without the *TERT* C228T mutation in NMU (*P* = 0.005; Fig. 3B). This association was not observed for the *TERT* C228T status in primary tumors (*P* = 0.265; Fig. 3C). When *TERT* C228T status in NMU was stratified by BCG instillation therapy, patients with *TERT* C228T mutation in NMU without BCG induction therapy had a significantly worse prognosis for bladder recurrence than patients with *TERT* C228T mutation with BCG therapy (*P* = 0.024; Fig. 3D). On the other hand, for patients without *TERT* C228T mutation in NMU, there was no difference in bladder recurrence between the patients with and without BCG therapy (Fig. 3E).

Finally, we performed univariate Cox proportional hazard analysis. The number of tumors [hazard ratio (HR): 3.1, 95% confidence interval (CI): 1.3–8.2] and the presence of the *TERT* C228T mutation in NMU (HR: 3.2, 95% CI: 1.3–7.6) were associated with bladder recurrence (Table 3). Multivariate Cox proportional hazard analysis revealed that the number of tumors (HR: 2.8, 95% CI: 1.2–7.5, *P* = 0.020) and the presence of the *TERT* C228T mutation in NMU (HR: 3.1, 95% CI: 1.3–7.6, *P* = 0.014) were significantly and independently associated with bladder recurrence (multivariate model 1). The *TERT* C228T mutation was also found to be an independent factor associated with bladder recurrence after adjustment for BCG instillation therapy, EORTC score, and age (*P* = 0.018; multivariate model 2).

**Table 3:** Univariate and multivariate analysis of factors associated with bladder recurrence

	Univariate			Multivariate model 1			Multivariate model 2		
	HR	95% CI	*P*‐value	HR	95% CI	*P*‐value	HR	95% CI	*P*‐value
Age (≥ 73 vs ≤ 72)	0.6	0.2–1.3	0.223				0.4	0.2–1.1	0.085
pT stage									
pT1 vs pTa	1.0	0.4–2.4	0.917						
CIS									
Positive vs negative	1.4	0.5–3.4	0.522						
Number of tumors									
Multiple vs solitary	3.1	1.3–8.2	0.010	2.8	1.2–7.5	0.020			
Size									
≥ 3 cm vs < 3 cm	2.0	0.6–5.4	0.250						
EORTC (≥ 5 vs ≤ 4)	2.1	0.9–5.0	0.142				1.9	0.7–5.6	0.227
2nd TUR (yes vs no)	0.9	0.3–2.1	0.871						
BCG (yes vs no)	0.9	0.3–2.2	0.873				0.4	0.1–1.1	0.064
*TERT* C228T in NMU‐positive vs urothelium‐negative	3.2	1.3–7.6	0.024	3.1	1.3–7.6	0.014	3.3	1.2–9.2	0.018

## 4 Discussion

In this proof‐of‐concept study, we report for the first time that the *TERT* C228T mutation was detected in NMU that was pathologically diagnosed as NU or UD, and that the mutation in NMU was associated with bladder recurrence after TURBT in patients with NMIBC. Therefore, analysis for the presence of the *TERT* C228T mutation in NMU may offer a more precise risk stratification of patients with NMIBC than do current stratifications. Furthermore, a positive result for the *TERT* C228T mutation in NMU might be a novel indication for BCG instillation therapy. Though certain mutational signatures are associated with patient age at cancer diagnosis [27], no difference was observed in age between patients with and without *TERT* C228T mutation in NMU.

Malignant tumors evolve from microscopic precursor lesions such as dysplasia; however, there may be cancer‐initiating cells with pathologically normal appearances in tumorigenesis as a consequence of field change caused by exposure to environmental carcinogens [21]. In bladder cancer, Majewski *et al.* [24] reported that DNA methylation was detected as one of the initial field changes, but little is actually known about the association between field change and *TERT* promoter mutations. To the best of our knowledge, there are currently no reports regarding clinical outcome for field change and malignant tumors.

Telomeres, which protect the ends of human chromosomes, are shortened by cell division during aging. TERT is a catalytic subunit of telomerase, which lengthens telomeres in DNA strands exceeding the Hayflick limit. In UC, the *TERT* C228T mutation is more frequently detected than the *TERT* C250T mutation [20]. Interestingly, the *TERT* C228T mutation increases *TERT* expression more than the *TERT* C250T mutation [16]. *TERT* promoter mutations may also be detected in urinary liquid biopsies from patients with no obvious evidence of cancer. When detected, this is associated with the development of UC. *TERT* promoter mutations overcome the proliferative barrier imposed by telomere shortening and thereby play a crucial role early in tumorigenesis by promoting immortalization and genomic instability. *TERT* promoter mutations are not detected in benign proliferative urothelial lesions [28]. Though these mutations are thought to be present in NMU during early stages of tumor formation prior to pathological change [29], the presence of these mutations has not been proven directly. Taken together, we hypothesize that *TERT* promoter mutations, especially *TERT* C228T, exist in urothelium that appears pathologically normal as a field change and play an important role in UC tumorigenesis.

EORTC has developed a scoring system and risk tables, which are widely recommended in various clinical guidelines [3, 5]. However, many researchers have reported that the EORTC risk tables cannot fully stratify risk for both disease recurrence and progression in external validation cohorts of patients with NMIBC [4, 6]. Therefore, a novel tool for improving the current risk stratification system is needed for patients with NMIBC.

There have been several reports regarding the molecular analysis of NMU of patients with UBC [30, 31, 32, 33]. Although field change in DNA methylation or loss of chromosome 9 heterogeneity may initiate bladder tumorigenesis, our study for the first time demonstrated an association between *TERT* promoter mutations and field change and also showed their clinical utility as prognostic biomarkers for bladder cancer recurrence.

The current study had several limitations, including its small sample size, its retrospective nature, and the fact that maintenance of BCG intravesical therapy was not administered. Because of its retrospective nature, the treatment strategy following TURBT varied, even in patients with similar clinical backgrounds. However, our results indicated that BCG instillation therapy may improve the recurrence rate of UBC after TURBT for patients with the *TERT* C228T mutation in NMU. Larger‐scale multi‐institutional and prospective studies are needed to validate this finding.

To diagnose concurrent CIS, we performed systematic random biopsies for all patients who had primary UBC and had received TURBT for the first time. However, it remains controversial whether random biopsies should be performed to identify CIS in mucosa that appears normal. An EORTC retrospective review found that 10% of random biopsy specimens were positive and proposed that when concurrent CIS is highly suspected, random biopsies are indicated only in instances of multiple tumors or positive cytology [34]. At our institution, the indication for random biopsy is widely applied to patients with UBC compared with that proposed by clinical guidelines. Our current findings demonstrated significant clinical utility of systematic random biopsies and their analysis for *TERT* promoter mutations. Although a prospective clinical trial is necessary, the analysis of *TERT* promoter mutation in NMU might be a novel indication for BCG instillation after TURBT, and the patients with *TERT* promoter mutation in NMU may benefit from BCG instillation for the prevention of bladder recurrence.

## 5 Conclusions

The *TERT* C228T mutation was detected in NU and UD of patients with NMIBC and may thus be a novel and useful prognostic factor for risk stratification of NMIBC. Analysis of systematic random biopsies for *TERT* promoter mutations in NMU may guide selection of the optimal treatment strategy for patients with NMIBC.

## Conflict of interests

The authors declare no conflict of interest.

## Author contributions

YH conceptualized the data, involved in formal analysis and methodology, investigated the study, and wrote the original draft. KF conceptualized the study, supervised the data, and wrote, reviewed, and edited the manuscript. SN and EM supervised and analyzed the data. ET, MM, KN, YK, CW, and YI investigated the data. TK, KH, AK, TU, MU, RI, and NN supervised the data.

## Supporting information

Table S1. ddPCR assay list.
