# Supplementary material for: TERT C228T mutation in non‐malignant bladder urothelium is associated with intravesical recurrence for patients with non‐muscle invasive bladder cancer
Source: Mol Oncol. 2020 Jun 27;14(10):2375–83. doi: 10.1002/1878-0261.12746 (PMC7530786; doi:10.1002/1878-0261.12746)
Supplement: Supplementary file 1 — Table S1. ddPCR assay list. [file MOL2-14-2375-s001.docx]

| **Supplementary Table S1: Droplet digital PCR assay list** | | | | | |
| --- | --- | --- | --- | --- | --- |
| Mutation Assay ID | Gene | Ref | Alt | Amplicon length (bp) |  |
| dHsaEXD72405942 | TERT | C | T | 113 |  |
| dHsaEXD46675715 | TERT | C | T | 113 |  |
